# A FAS -670A/G single nucleotide polymorphism may be associated with HTLV-1 infection and clinical evolution to TSP/HAM

**DOI:** 10.1186/1742-4690-8-S1-A63

**Published:** 2011-06-06

**Authors:** Antonio C R Vallinoto, Bárbara B Santana, Ethienne Lobato dos Santos, Rafaela Resplande do Espírito Santo, Renata Bezerra Hermes, Rita C Medeiros Sousa, Izaura M V Cayres Vallinoto, Luiz F A Machado, Marluísa O G Ishak, Ricardo Ishak

**Affiliations:** 1 Laboratório de Virologia, Instituto de Ciências Biológicas, Universidade Federal do Pará, Belém, PA, Brazil; 2 Hospital Universitário João de Barros Barreto, Belém, PA, 66075-900, Brazil

## 

FAS and FASL genes are closely linked to the mechanism of the immune system and several polymorphisms in these genes have been associated with susceptibility to diseases. This study investigated the polymorphisms at positions -670 in the FAS gene, and -169 (IVS3nt169) and -124 (IVS2nt-124) in the FASL gene, among HTLV-1/2 infected subjects. Blood samples from 66 HTLV infected subjects and 192 seronegative individuals were collected, and polymorphisms were analyzed using a polymerase chain reaction (PCR) followed by RFLP analysis using restriction endonucleases. The products were visualized after electrophoresis in 4% agarose gel. The genotype frequencies of the FAS -670 polymorphism showed a higher prevalence of genotype -670GG among HTLV-1 infected subjects (46.97%) as compared to the control group (30.73%; p=0.0162). The comparative analysis between symptomatic and asymptomatic HTLV infected subjects showed a higher prevalence (p=0.0007) of the genotype -670GG among patients with TSP/HAM (50%) as compared to asymptomatic subjects (45%). TCD4+ and TCD8+ lymphocyte counts from HTLV infected and seronegative subjects were compared but no significant association was observed. The proviral load values according to the status of symptomatic and asymptomatic infection carrying different genotypes were compared but showed no statistical significance. The present results suggest that FAS -670 polymorphism seems to be associated with susceptibility to HTLV-1 and may increase the chance to develop TSP/HAM among HTLV-1 infected persons.

